# J. H. Parry

**Published:** 1906-06

**Authors:** 


### Obituary Notice.

---

### J. H. PARRY, L.R.C.P. and S. Edin.

We regret to note the death of Mr. J. H. Parry, at the age of 56. He was a student of the Bristol Medical School, and at the General Hospital gained the Clarke Surgical Scholarship. He obtained the diplomas of L.R.C.P. and S. Edin. in 1877, and was for many years a busy and successful practitioner, and until recently was Medical Officer of Health for the Horfield District Council. In 1892 he was President of the Bristol Cambrian Society.

